# “Brain drain” amongst Israeli physicians who graduated abroad

**DOI:** 10.1186/s13584-025-00720-5

**Published:** 2025-10-29

**Authors:** Tomer Swechinsky, Rachel Berner-Shalem

**Affiliations:** 1 Strategic and Economic Planning Administration, Israeli Ministry of Health, Jerusalem, Israel; 2 Azrieli Faculty of Medicine, Bar-Ilan University, Zfat, Israel

**Keywords:** Medical workforce, Brain drain, Israeli physician shortage

## Abstract

**Background:**

Israel relies heavily on foreign-trained physicians, with approximately 58% of all its practicing physicians having received their medical education abroad. The Israeli Ministry of Health (MOH) has estimated a 30% non-return rate (i.e., the proportion of Israeli medical graduates who remain abroad after completing their studies, rather than returning to practice in Israel) among Israeli medical graduates studying abroad. However, this estimate is based on limited data. This study explores the self-reported class non-return rate among Israeli medical graduates from foreign medical schools and examines potential factors influencing their decision to remain abroad.

**Methods:**

This study analyzed data provided by the Israeli MOH regarding Israeli medical graduates who completed their education at foreign medical schools accredited under the Yatziv Reform between 2023 and 2024. The data were collected via a structured online survey developed by the MOH in 2024–2025 and distributed through WhatsApp. Respondents provided self-reported information including: university attended, year of graduation, number of Israeli students in the cohort (hereafter referred to as class size), and the number of Israelis reported to have returned to practice in Israel. The primary outcome was the self-reported non-return rate at the class level.

**Results:**

Among 101 valid responses representing 37 classes and approximately 1,048 Israeli medical graduates across 11 countries, the overall non-return rate was 9.7%, with substantial variation by country, from 0% in Jordan to 40.5% in Italy. Excluding graduates from the Palestinian Authority (PA) increased the rate to 12.1%, though not significantly. A significant negative correlation was found between class size and non-return rate (ρ = − 0.51, *p* = 0.0011), while higher Human Development Index (HDI) values were associated with both smaller class sizes (ρ = − 0.72, *p* < 0.001) and higher non-return rates (ρ = 0.53, *p* = 0.0007). A multiple regression model with class size, HDI, and their interaction explained 31% of the variance in non-return rates (Adj. R² = 0.31; *p* = 0.002), with all terms significant.

**Conclusions:**

The self-reported non-return rate was lower than expected but varied substantially by country. Class size, HDI, and their interaction were significant predictors of non-return, highlighting the need for targeted return policies and improved data to inform workforce planning.

## Background

### Global trends in foreign medical education and physician migration

Medical education abroad has become increasingly common due to limited domestic capacity, competitive admission requirements, and growing global demand for healthcare professionals [1–3]. Countries such as Israel, New Zealand, Ireland, and Norway rely heavily on internationally trained physicians to meet workforce needs [4]. For instance, New Zealand faces a severe doctor shortage, with over 40% of its physician workforce trained abroad, the second highest proportion among OECD countries, following Israel. Many New Zealand-trained doctors emigrate, particularly to Australia, due to lower pay, burnout, and limited postgraduate opportunities at home, raising concerns about sustainability and retention [5]. Similar challenges have been documented elsewhere. Studies in Ireland and Croatia revealed high intentions among medical students to migrate abroad after graduation, citing poor working conditions and limited training opportunities as influencing factors [6, 7]. In Sub-Saharan Africa, large-scale physician emigration has raised urgent concerns about workforce sustainability in countries facing healthcare access challenges [8]. A study of China-educated international medical students - mostly from Asian and African countries - found that less than half intended to return to their home countries, with higher academic performance and female gender associated with preferences for migration to high-income countries [9]. Collectively, these trends underscore the global nature of medical migration and the complex interplay of personal aspirations and structural limitations across diverse regions.

### The situation in Israel

Israel has the highest rate of foreign-trained physicians among OECD countries, with approximately 58% of all practicing physicians having received their medical education abroad [4, 10]. This reliance is largely due to the limited capacity of Israeli medical schools, which admit far fewer students than necessary [11, 12]. The only currently available estimate by the Ministry of Health suggests that approximately 30% of Israeli medical students who study abroad do not return to practice in Israel [3]; however, this figure is based on unpublished internal data and should be interpreted with caution due to unclear methodology and representativeness.

Moreover, Israel’s case is unique in that most of its foreign-trained physicians are native Israelis who deliberately pursue medical education abroad. With limited data existing, the MOH established a voluntary registry for Israeli medical students abroad to improve data collection and workforce planning. However, participation remains incomplete, and the registry does not yet capture the full scope of the student population [3]. Earlier studies of Israel’s physician workforce also highlighted this gap, emphasizing the importance of linking registry and employment data to accurately assess physician availability and outflow [13].

One response to these workforce and regulatory challenges has been creating programs that enable Israeli medical students abroad to return to Israel during the clinical years of their training to complete rotations in local hospitals [14]. These placements, often facilitated through agreements with foreign universities, allow for early integration into the Israeli healthcare system. Recognizing their potential, the Ministry of Health issued a directive [15] to encourage but also to regulate these rotations, ensuring they do not overburden the clinical training capacity reserved for students in Israeli medical schools.

### Factors influencing return behavior

Return decisions are shaped by “push” and “pull” factors [16]. Push factors refer to conditions that drive individuals away from their home country, while pull factors are conditions in the destination country that attract individuals. According to the MOH, most Israeli students study in Eastern Europe, Jordan, and the Palestinian Authority (PA) [17] - countries that vary significantly in cost of living, training quality, and Human Development Index (HDI) rankings [18]. These factors may serve as push and/or pull factors that influence students’ likelihood of returning. Notably, institutions located in the PA may represent a unique context due to their geographic and social proximity to Israel, which could shape students’ experiences and post-graduation decisions in distinct ways.

In addition to these structural considerations, recent work further underscores the relevance of professional and career-related factors, showing that financial security, fellowship opportunities, and dedicated time for research and teaching strongly shape return decisions [19].

### Aim of the study

This study aims to assess the self-reported non-return rates of Israeli medical students who studied abroad and to examine country- and faculty-level patterns in non-return rates, in order to inform workforce planning and policy development.

## Methods

### Study design and participants

This study analyzed data provided by the Financial and Strategic Planning Administration at the Israeli MOH regarding Israeli physicians who graduated in 2023 and 2024 from medical schools abroad accredited under the Yatziv Reform. The dataset contained self-reported information about the non-return rate, defined as the proportion of Israeli graduates who were declared not to return to practice in Israel.

Data were collected through a survey developed and conducted by the MOH in 2024–2025. Respondents were contacted primarily via WhatsApp using contact details from the voluntary registry of Israeli medical students abroad that was established by the MOH in 2022. Participation was voluntary, and completion of the questionnaire was not mandatory.

**Inclusion and Exclusion Criteria**: The analysis included only individuals who met all of the following conditions: (1) graduated in 2023 or 2024; (2) were Israeli citizens; (3) studied at foreign medical schools accredited under the Yatziv Reform; and (4) provided a complete response to the questionnaire. Respondents or institutions not fulfilling all four criteria were excluded from the analysis.

### Analyzed data

The analyzed data collected by the MOH included the following information from participants:

1. Faculty name (e.g., *Università di Bologna*,* Italy*).
2. Graduation year (2023 or 2024).
3. Class size (Number of Israeli classmates in their cohort) according to the respondent’s best knowledge.
4. Number of Israeli classmates who returned to Israel to practice medicine, according to the respondent’s best knowledge.

### Data validation

To enhance data reliability:

- The MOH sought multiple responses from the same class when possible. In classes with more than 10 Israeli students, the MOH made efforts to gather responses from more than one participant to verify the data.
- Outliers and discrepancies in responses, such as conflicting reports about the number of Israeli students in a class or returnees to Israel, were reviewed by the MOH. When substantial differences were identified, the MOH attempted to recontact the respondents for clarification. Minor inconsistencies that did not meaningfully affect class-level medians were retained. If discrepancies remained unresolved after follow-up, those responses were excluded to maintain data integrity.
- Survey responses were not cross-validated with other data sources.

### Human development index (HDI)

The Human Development Index (HDI) was used as a proxy for national living standards. HDI values for each country were obtained from the United Nations Development Programme (UNDP) 2023–2024 Human Development Report [18] and were assigned accordingly to explore potential associations with the non-return rate. While not based on individual-level data, HDI served as a country-level contextual factor.

### Statistical analysis

Data analysis was performed using R version 4.3.0. Class size was defined as the number of Israeli students in a given cohort (faculty × year). When multiple responses were available for the same class, the reported values were aggregated at the class level by computing the median across respondents. The same approach was applied to the number of Israeli returnees per class. These class-level medians were then used to estimate the median non-return rate per class. This method allowed for consistent aggregation while accounting for variability and potential outliers in self-reported data. For each class-level variable (class size, number of returnees, and non-return rate), interquartile ranges (IQRs) were computed and reported as the 25th–75th percentile (Q1–Q3), wherever applicable. At the country level, IQRs represent the distribution of faculty-level median values, reflecting variation across faculties within each country. At the total level, class size and non-return rates were derived from the sum of faculty-level medians. Because these are aggregate values rather than sample distributions, no formal IQR or other dispersion measures were calculated at this level, with the exception of the non-return rate, for which we report the IQR of class-level medians across the dataset to provide a visual summary of dispersion.

The normality of the non-return rate was tested using the Shapiro-Wilk test. Since the data did not follow a normal distribution, non-parametric methods were employed.

Spearman’s rank correlations were calculated to examine the relationships between: (1) HDI and median class size, (2) HDI and median non-return rate, and (3) median class size and non-return rate. A multiple linear regression model was constructed using median class size, HDI, and their interaction as predictors of the non-return rate across countries, based on class-level median values.

Finally, a sensitivity analysis was conducted to test the impact of including or excluding data from PA institutions. Return rates were recalculated with and without PA graduates to assess their influence on national-level estimates.

## Results

### Response rate and data representation

**Fig. 1:**
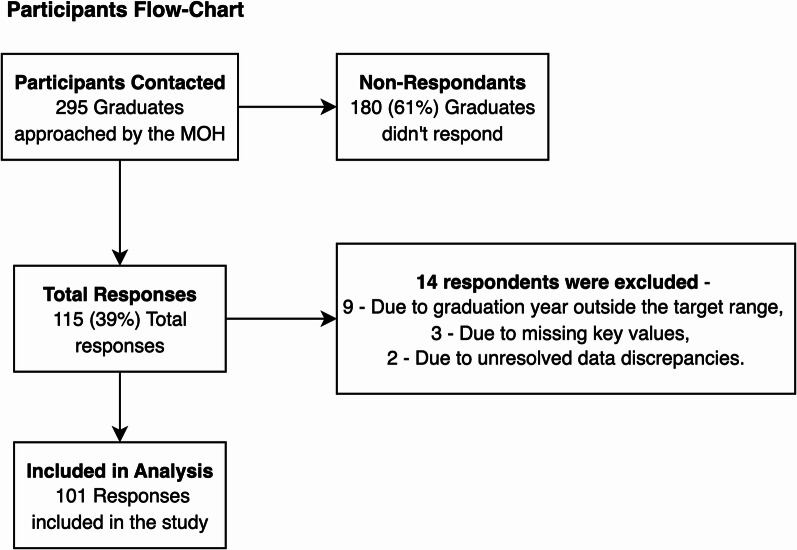
Flow Diagram of Survey Response and Inclusion Process. Flowchart of participant inclusion and exclusion. The diagram outlines the processing of survey responses and resulting analytic sample used in the study

A total of 295 graduates were contacted by the MOH, with 115 (38.9%) submitting responses. After excluding entries not meeting the inclusion criteria, 101 responses were included in the final analysis (Fig. 1). These responses represent 37 classes across 11 countries. Based on self-reported estimates from respondents, these classes collectively reported a total of 1,048.5 graduates between 2023 and 2024, calculated as the sum of the median class sizes reported per class. Respondents represented a diverse range of Yatziv-accredited institutions across Central and Eastern Europe and the Middle East. A detailed breakdown of countries and faculties is provided in Table 1.

**Table 1:** Class size and response data for Israeli medical graduates abroad, by faculty and graduation yea

Location	Faculty	Year of Graduation	Median Class Size (Q1–Q3)	Respondents
Croatia	University of Zagreb	2023	12.0 (12.0–12.0)	2
Croatia	University of Zagreb	2024	16.0 (16.0–16.8)	6
Czech Republic	Charles University, First Faculty	2023	20.0 (20.0–20.0)	2
Czech Republic	Charles University, First Faculty	2024	14.0 (14.0–14.5)	7
Czech Republic	Masaryk University	2023	8.0 (8.0–8.0)	1
Czech Republic	Masaryk University	2024	9.0 (8.0–9.5)	3
Czech Republic	Palacký University	2024	14.0 (14.0–14.0)	5
Hungary	Semmelweis University	2024	17.5 (13.8–20.5)	4
Hungary	University of Debrecen	2023	27.0 (25.0–30.0)	5
Hungary	University of Debrecen	2024	33.5 (32.0–35.5)	4
Hungary	University of Szeged	2023	15.0 (14.0–17.5)	4
Hungary	University of Szeged	2024	9.0 (9.0–9.0)	1
Italy	Humanitas University, Milan	2023	5.0 (5.0–5.0)	1
Italy	University of Bologna	2023	5.0 (5.0–5.0)	1
Italy	University of Bologna	2024	5.0 (5.0–5.0)	1
Italy	University of Pavia	2023	7.5 (6.8–8.2)	2
Italy	University of Turin	2023	10.0 (9.5–10.5)	2
Italy	University of Turin	2024	6.0 (6.0–6.0)	2
Italy	Università di Brescia	2023	2.0 (2.0–2.0)	1
Jordan	Jordan university of Science and technology	2023	55.0 (45.0–70.0)	3
Jordan	Jordan university of Science and technology	2024	35.0 (35.0–35.0)	1
Lithuania	Lithuanian University of Health Sciences (LSMU)	2023	70.0 (70.0–100.0)	5
Lithuania	Lithuanian University of Health Sciences (LSMU)	2024	75.0 (63.8–81.8)	8
Lithuania	Vilnius University	2023	8.0 (7.5–8.0)	3
Lithuania	Vilnius University	2024	3.0 (3.0–3.0)	1
Palestinian Authority	Al-Quds University	2023	55.0 (55.0–55.0)	1
Palestinian Authority	Al-Quds University	2024	65.0 (65.0–65.0)	1
Palestinian Authority	An-Najah National University	2023	70.0 (70.0–70.0)	1
Palestinian Authority	An-Najah National University	2024	105.0 (90.0–130.0)	5
Poland	Medical University of Silesia, Katowice	2023	37.5 (36.2–38.8)	2
Poland	Medical University of Silesia, Katowice	2024	28.0 (27.0–29.0)	5
Poland	University of Warsaw	2024	6.5 (6.2–6.8)	2
Romania	Carol Davila University of Medicine	2023	63.0 (62.0–64.0)	2
Romania	Carol Davila University of Medicine	2024	90.0 (90.0–95.0)	3
Romania	University of Cluj-Napoca	2024	10.0 (10.0–10.0)	1
Slovakia	Pavol Jozef Šafárik University (UPJS)	2023	12.0 (12.0–12.0)	1
Slovakia	Pavol Jozef Šafárik University (UPJS)	2024	25.0 (22.5–27.5)	2
Total		2023–4	1048.5	101

Each row represents a cohort of Israeli medical students by faculty and year of graduation. “Median Class Size” reflects the median number of Israeli students reported for that class, with interquartile range (IQR = Q1–Q3) indicating dispersion. “Respondents” indicates the number of survey participants who contributed data for each class. The total class size is the sum of class-level medians

### Overall non-return rates

The non-return rate for Israeli medical graduates was calculated by summing the total number of graduates and the number of those who were declared to have returned to Israel across all 37 classes. Based on these totals, the overall non-return rate was 9.7% (Q1-Q3 = 0–40), indicating considerable variability in non-return rates across classes.

Class sizes varied across countries and programs. For example, the University of Zagreb in Croatia had class sizes of 12 (Q1–Q3 = 12–12) in 2023 and 16 (Q1–Q3 = 16–16.8) in 2024, while larger classes were seen at An-Najah National University (class size = 105, Q1–Q3 = 90–130) in 2024 (Table 1).

The non-return rates varied significantly between countries. Places like Jordan and the PA had lower non-return rates, at 0% (Q1–Q3 = 0–0) and 3.4% (Q1–Q3 = 0–4.6), while Italy and the Czech Republic had higher non-return rates, at 40.5% (Q1–Q3 = 39.2–41.7) and 29.2% (Q1–Q3 = 14.3–42.9) (Table 2).

**Table 2:** Class-Level and Country-Level Non-Return rates for Israeli medical graduates

Location	Faculty	Year of Graduation	Graduates Declared to Have Returned (Q1–Q3)	Non-Return Rate (%) (Q1–Q3)	Non-Return Rate (%) (Q1-Q3 of Class Medians)
Croatia	University of Zagreb	2023	11.5 (11.2–11.8)	4.2 (2.1–6.3)	8.9 (6.9–12.4)
	University of Zagreb	2024	14.0 (14.0–14.0)	15.1 (12.5–18.5)	
Czech Republic	Charles University, First Faculty	2023	10.5 (10.2–10.8)	47.5 (46.2–48.8)	29.2 (14.3–42.9)
	Charles University, First Faculty	2024	12.0 (12.0–12.5)	14.3 (13.8–14.3)	
	Masaryk University	2023	8.0 (8.0–8.0)	0.0 (0.0–0.0)	
	Masaryk University	2024	7.5 (6.8–7.8)	16.7 (15.5–18.3)	
	Palacký University	2024	8.0 (7.0–8.0)	42.9 (41.7–42.9)	
Hungary	Semmelweis University	2024	14.5 (11.0–18.0)	17.5 (12.4–20.0)	9.8 (5–17.5)
	University of Debrecen	2023	25.0 (23.0–28.0)	5.0 (0.0–6.7)	
	University of Debrecen	2024	33.5 (32.0–35.5)	0.0 (0.0–0.0)	
	University of Szeged	2023	13.0 (10.8–16.2)	10.0 (0.0–23.3)	
	University of Szeged	2024	6.0 (6.0–6.0)	33.3 (33.3–33.3)	
Italy	Humanitas University, Milan	2023	1.0 (1.0–1.0)	80.0 (80.0–80.0)	45.7 (42.5–65)
	University of Bologna	2023	3.0 (3.0–3.0)	40.0 (40.0–40.0)	
	University of Bologna	2024	1.0 (1.0–1.0)	80.0 (80.0–80.0)	
	University of Pavia	2023	7.5 (6.8–8.2)	0.0 (0.0–0.0)	
	University of Turin	2023	5.5 (5.2–5.8)	44.9 (44.7–45.2)	
	University of Turin	2024	3.0 (3.0–3.0)	50.0 (50.0–50.0)	
	Università di Brescia	2023	1.0 (1.0–1.0)	50.0 (50.0–50.0)	
Jordan	Jordan university of Science and technology	2023	55.0 (45.0–70.0)	0.0 (0.0–0.0)	0 (0–0)
	Jordan university of Science and technology	2024	35.0 (35.0–35.0)	0.0 (0.0–0.0)	
Lithuania	Lithuanian University of Health Sciences (LSMU)	2023	60.0 (60.0–100.0)	14.3 (3.3–14.3)	13.1 (5–25)
	Lithuanian University of Health Sciences (LSMU)	2024	69.5 (60.5–75.5)	6.7 (5.8–8.5)	
	Vilnius University	2023	3.0 (3.0–4.0)	57.1 (47.3–59.8)	
	Vilnius University	2024	3.0 (3.0–3.0)	0.0 (0.0–0.0)	
Palestinian Authority	Al-Quds University	2023	45.0 (45.0–45.0)	18.2 (18.2–18.2)	3.4 (0–4.6)
	Al-Quds University	2024	65.0 (65.0–65.0)	0.0 (0.0–0.0)	
	An-Najah National University	2023	70.0 (70.0–70.0)	0.0 (0.0–0.0)	
	An-Najah National University	2024	105.0 (90.0–125.0)	0.0 (0.0–3.8)	
Poland	Medical University of Silesia, Katowice	2023	37.5 (36.2–38.8)	0.0 (0.0–0.0)	4.6 (0–25)
	Medical University of Silesia, Katowice	2024	28.0 (27.0–29.0)	0.0 (0.0–0.0)	
	University of Warsaw	2024	3.2 (3.1–3.4)	50.0 (50.0–50.0)	
Romania	Carol Davila University of Medicine	2023	58.5 (58.2–58.8)	7.1 (6.0–8.2)	7.1 (3.5–7.4)
	Carol Davila University of Medicine	2024	83.0 (79.0–89.0)	7.8 (6.4–12.2)	
	University of Cluj-Napoca	2024	10.0 (10.0–10.0)	0.0 (0.0–0.0)	
Slovakia	Pavol Jozef Šafárik University (UPJS)	2023	10.0 (10.0–10.0)	16.7 (16.7–16.7)	16.2 (16–16.5)
	Pavol Jozef Šafárik University (UPJS)	2024	21.0 (19.0–23.0)	15.8 (15.4–16.2)	
Total		2023–4	947.2	9.7 (0.0–40.0)	

Each row presents a class-level estimate of the number of Israeli graduates reported to have returned to Israel (median, IQR), the corresponding class-level non-return rate (%), and the aggregated country-level non-return rate (%). Country-level IQRs reflect the interquartile range (Q1–Q3) of faculty-level median non-return rates. For the total row, the non-return rate is calculated from the sum of class medians, and the IQR represents the Q1–Q3 range across the class-level medians

We conducted a sensitivity analysis to assess the impact of including PA graduates on the overall results. When PA graduates were excluded, the national non-return rate increased from 9.7 to 12.1%. This suggests that the inclusion of PA responses affects the national estimate, and should be considered when interpreting the overall rate.

### Class size and non-return tendencies

**Fig. 2:**
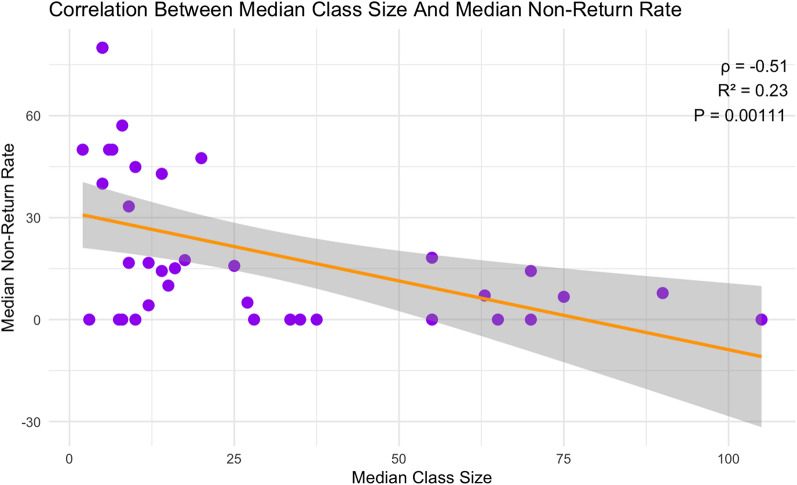
Correlation Between Median Class Size and Non-Return Rate. This scatter plot illustrates the relationship between median class size and the median non-return rate. A negative correlation is observed, with larger classes tending to have lower non-return rates. The orange curve represents the smoothed trend, while the shaded area indicates the confidence interval

A significant negative correlation was observed between class size and non-return rate (ρ = −0.51, *p* = 0.0011). Larger classes exhibited lower non-return rates compared to smaller classes (Fig. 2).

### Correlations with HDI

**Fig. 3:**
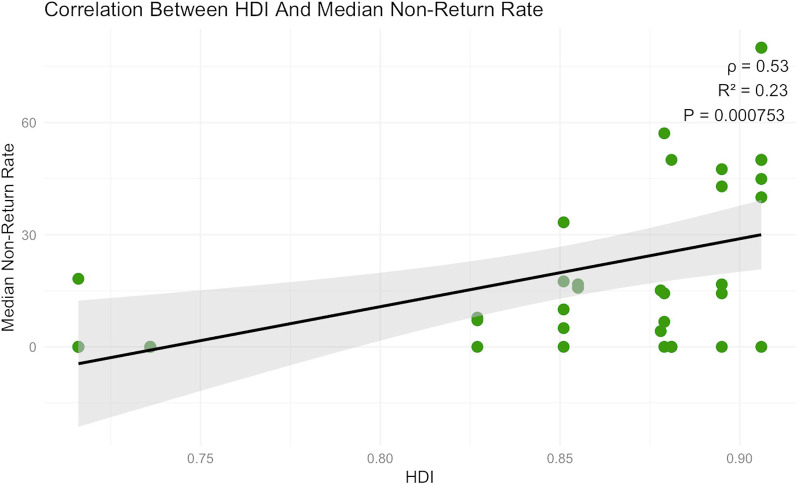
Correlation Between Human Development Index (HDI) and Median Non-Return Rate. Scatterplot showing the association between country-level Human Development Index (HDI) and the median non-return rate of Israeli medical graduates. Each point represents one class. A positive correlation was observed (Spearman's ρ = 0.53, R² = 0.23, p = 0.00075), indicating that higher HDI values are associated with higher non-return rates. The black line represents the linear regression fit with 95% confidence intervals (shaded area)

HDI was assigned to each country in the dataset to explore contextual factors. A significant negative correlation was found between HDI and class size (ρ = −0.72, *p* < 0.001), while a positive correlation was observed between HDI and non-return rate (ρ = 0.53, *p* = 0.0007) (Fig. 3).

### Multiple regression analysis

A multiple linear regression using class-level medians found that median class size, HDI, and their interaction were all statistically significant predictors of non-return rates (*p* = 0.047, *p* = 0.011, and *p* = 0.035, respectively). The model accounted for 31% of the variance in non-return rates (adjusted R² = 0.31; *p* = 0.002). These findings reflect measurable associations between class size, HDI, and variation in declared return outcomes across countries.

## Discussion

### Non-return rate findings

Our study identified a 9.7% non-return rate among Israeli medical graduates abroad for the graduating classes of 2023–2024, substantially lower than the cited 30% estimate by the Israeli MOH [3]. While this finding reflects a specific snapshot in time and may not represent earlier or future trends, it reveals a significant gap that underscores the limitations of intention-based forecasts.

Comparative international data also suggest that the Israeli case may be relatively unique. In Canada, only 22.6% of Canadian citizens who studied medicine abroad were able to secure a residency position in Canada [20], implying a non-return rate of over 75%. A longitudinal mixed-methods study of international medical program graduates across Europe found that only around 40% had returned to their home country two years after graduation [21], while approximately 32% had relocated to a third country. Similarly, a study of China-educated international medical students—primarily from low- and middle-income countries—found that fewer than half intended to return to their country of origin [9].

These findings suggest that Israeli medical students abroad demonstrate comparatively high return rates, potentially driven by factors such as national identity, geographic proximity, and regulatory pathways. While a 9.7% non-return rate is not negligible, it is modest by international standards. This reinforces the argument that broad, undifferentiated return incentive programs may yield limited results. Instead, more targeted strategies—tailored to specific populations, countries of study, and regional workforce needs—may prove more effective in addressing physician shortages in Israel.

### Class size correlation

A negative correlation was observed between class size and non-return rates. One possible explanation is that students in smaller classes are more likely to integrate into local communities - for example, by finding a local partner or job - while larger groups of Israelis abroad may reinforce social ties with fellow nationals, making return more likely [22]. While this relationship does not imply causation, it offers a new lens for interpreting return patterns.

### Different countries show different tendencies of returning

Declared return rates varied by country. Graduates from Jordan and the PA had the lowest non-return rates, likely due to geographic proximity to Israel and additional systemic factors. In Jordan’s case, factors may include a long-standing culture of medical emigration, with over a third of Jordanian-trained physicians working abroad [22]. In contrast, Italy and the Czech Republic had higher non-return rates. In Italy, smaller class sizes may support greater integration into local communities [23]. The Czech Republic’s high HDI may encourage graduates to remain [18].

Notably, Poland exhibited low non-return rates among Israeli graduates despite its relatively high Human Development Index (HDI), which may reflect stronger national infrastructure. This apparent contradiction could be explained by systemic challenges within the Polish healthcare system, such as excessive workload, limited specialization opportunities, and weak support for professional development. These same factors have been identified as drivers of emigration among Polish healthcare workers themselves [24] and may similarly discourage Israeli graduates from remaining after graduation.

### Considering the unique context of the Palestinian authority

The unique geographic and social proximity of PA institutions to Israel underscores the importance of considering localized dynamics when interpreting return behaviors, as these settings may shape students’ decisions in ways not captured by broader international comparisons.

### Correlations with HDI and multivariable modeling

We found that HDI was positively correlated with non-return rates and negatively correlated with class size, indicating that students trained in countries with higher living standards may be more inclined to remain abroad. In our multivariable regression model, both HDI and class size, as well as their interaction, were statistically significant predictors of non-return. The interaction term suggests that the association between class size and non-return varies by national development level. The model explained 31% of the variance in non-return rates (adjusted R² = 0.31), highlighting the relevance of both educational and contextual national characteristics.

Another possible explanation for higher non-return in high-HDI countries is the relative ease of combining clinical practice with research abroad, supported by protected research time and funding in systems such as the U.S [25, 26]. Although Israel has begun to develop pathways for clinician-scientists, such as structured research training programs for family medicine residents [27], these opportunities remain limited and may partly explain observed patterns, as also shown among Israeli physicians in fellowship programs abroad [19].

### Policy proposals

1. **Increase Professional Support**: Given the relatively low number of Israeli graduates who remain abroad, policy efforts may be more effective if focused on supporting students during their studies. Initiatives such as webinars, academic mentoring, and clinical preparation programs can help maintain ties to Israel and strengthen graduates’ readiness to return and contribute to the Israeli healthcare system.
2. **Promoting Clinical Training in Underserved Regions**: Expanding opportunities for Israeli students abroad to complete clinical rotations in Israel, especially in underserved areas like the Negev and Galilee, may encourage return and long-term service. These placements, regulated by a Ministry of Health directive (*14*, *15*) are typically arranged through agreements with foreign medical schools. The directive highlights their potential to support reintegration, while also aiming to protect local training capacity. However, no empirical evidence currently evaluates whether such rotations increase return rates—a question that warrants further study. Nevertheless, exposure to the Israeli health system, particularly in specialties with available training capacity (e.g., family medicine, dermatology, and radiology), may align with national workforce goals and warrants further exploration.
3. **Support Student Enrollment in High-Quality Faculties with Larger Class Sizes**: The observed negative correlation between class size and non-return rate suggests that larger Israeli classes may be linked to higher return rates, possibly due to reduced integration abroad. While causality cannot be established, encouraging enrollment in reputable faculties that already attract larger groups of Israeli students may indirectly support return rates. This recommendation is exploratory and merits further research.
4. **Develop a Voluntary National Registry of Israeli Medical Students Abroad**: Accurate workforce planning requires better data on Israelis studying medicine abroad. As current systems are fragmented and incomplete, we propose a stepwise strategy to establish a voluntary national registry. In its initial phase, Israeli students would be incentivized to register with the MOH—e.g., through access to subsidized clinical rotations or reduced national insurance contributions. In parallel, we suggest incorporating simple return-tracking questions (e.g., year and country of graduation, class size and number of returned graduates) into existing onboarding forms during the internship year. Together, these measures would provide a sustainable foundation for future policy planning without deterring potential returnees.

While the proposed registry would not capture graduates who never engaged with the Israeli system, periodic reviews could help identify students who once registered but ultimately did not return—offering a potential future target for outreach efforts.

5. **Improving Routine Data Collection**: To support ongoing monitoring, we suggest the MOH add brief return-tracking questions (e.g., year and country of graduation, class size) to existing onboarding forms for new interns. This low-burden addition could enable sustainable, routine data collection. While based on peer-reported data, this study offers a practical model for estimating return rates and informing future tracking and targeted incentive efforts.

By adopting these proposals, Israel can better support medical students abroad, increase return rates, and strengthen the healthcare system through the reintegration of well-trained graduates.

### Limitations

This study provides new insights but has several limitations. First, all data were peer-reported by graduates and may be influenced by non-response, recall or social desirability bias. Although multiple responses per class were used to improve accuracy, the non-return rate reflects declared observed behavior, not personal intention.

Second, due to the lack of a comprehensive registry of Israeli medical students abroad, self-reported data was the most feasible source. The recent voluntary MOH registry remains incomplete, limiting generalizability.

Third, the final sample—101 respondents—represents less than 10% of the estimated 2023–2024 graduating population across the included schools, constraining the representativeness of findings.

Fourth, the survey was conducted during a period of significant national events, including judicial reform debates and the October 7 war, which may have influenced return decisions. However, the sample size per graduation year was too small to assess changes before and after these events.

Fifth, important individual-level variables such as demographics, finances, political views, or career considerations (e.g., opportunities for protected research time) were not assessed. While national HDI scores were used to reflect broader context, this study cannot establish causality.

Finally, Palestinian Authority institutions represent a unique case: many students studying there reside in Israel, complicating comparisons with other countries. Excluding PA classes raised the national non-return rate from 9.7 to 12.1%, though these responses were retained to reflect their growing share of Israeli graduates abroad.

## Conclusion

This study found that the peer-reported non-return rate of Israeli medical graduates abroad is considerably lower than previously estimated, though patterns vary across countries and are associated with factors such as class size and national living standards. These insights highlight the importance of targeted, data-driven strategies to strengthen connections with Israeli students abroad, support their reintegration into the domestic healthcare system, and guide future research aimed at better understanding the multilevel drivers of return behavior.

## Data Availability

No datasets were generated or analysed during the current study.

## Abbreviations

MOH: Ministry of Health
PA: Palestinian Authority
HDI: Human Development Index
Class size (Israeli class): Refers specifically to the number of Israeli students enrolled in a given faculty and graduation year abroad. This does not refer to the total number of students in the international program
